# Cenicriviroc, a Novel CCR5 (R5) and CCR2 Antagonist, Shows In Vitro Activity against R5 Tropic HIV-2 Clinical Isolates

**DOI:** 10.1371/journal.pone.0134904

**Published:** 2015-08-06

**Authors:** Benoit Visseaux, Charlotte Charpentier, Gilles Collin, Mélanie Bertine, Gilles Peytavin, Florence Damond, Sophie Matheron, Eric Lefebvre, Françoise Brun-Vézinet, Diane Descamps, ANRS CO5 HIV-2 Cohort

**Affiliations:** 1 INSERM, IAME, UMR 1137, Paris, France; 2 Univ Paris Diderot, IAME, UMR 1137, Sorbonne Paris Cité, Paris, France; 3 AP-HP, Hôpital Bichat, Laboratoire de Virologie, Paris, France; 4 AP-HP, Hôpital Bichat, Pharmacologie des antirétroviraux, Paris, France; 5 AP-HP, Hôpital Bichat, Service des Maladies Infectieuses et Tropicales, Paris, France; 6 Tobira Therapeutics, Inc., South San Francisco, CA, United States of America; University of Pittsburgh Center for Vaccine Research, UNITED STATES

## Abstract

**Background:**

Maraviroc activity against HIV-2, a virus naturally resistant to different HIV-1 antiretroviral drugs, has been recently demonstrated. The aim of this study was to assess HIV-2 susceptibility to cenicriviroc, a novel, once-daily, dual CCR5 and CCR2 antagonist that has completed Phase 2b development in HIV-1 infection.

**Methods:**

Cenicriviroc phenotypic activity has been tested using a PBMC phenotypic susceptibility assay against four R5-, one X4- and one dual-tropic HIV-2 clinical primary isolates. All isolates were obtained by co-cultivation of PHA-activated PBMC from distinct HIV-2-infected CCR5-antagonist-naïve patients included in the French HIV-2 cohort and were previously tested for maraviroc susceptibility using the same protocol. HIV-2 tropism was determined by phenotypic assay using Ghost(3) cell lines.

**Results:**

Regarding the 4 R5 HIV-2 clinical isolates tested, effective concentration 50% EC_50_ for cenicriviroc were 0.03, 0.33, 0.45 and 0.98 nM, similar to those observed with maraviroc: 1.13, 0.58, 0.48 and 0.68 nM, respectively. Maximum percentages of inhibition (MPI) of cenicriviroc were 94, 94, 93 and 98%, similar to those observed with maraviroc (93, 90, 82, 100%, respectively). The dual- and X4-tropic HIV-2 strains were resistant to cenicriviroc with EC50 >1000 nM and MPI at 33% and 4%, respectively.

**Conclusions:**

In this first study assessing HIV-2 susceptibility to cenicriviroc, we observed an *in vitro* activity against HIV-2 R5-tropic strains similar to that observed with maraviroc. Thus, cenicriviroc may offer a once-daily treatment opportunity in the limited therapeutic arsenal for HIV-2. Clinical studies are warranted.

## Introduction

Human Immunodeficiency Virus type 2 (HIV-2) was discovered few years after HIV type 1 (HIV-1). Currently, between one and two millions of people are estimated to be infected with HIV-2. HIV-2 is mainly present in West Africa including Guinea-Bissau, Gambia, Senegal, and Guinea. HIV-2 is also prevalent in some European countries, mainly in Portugal and France. HIV-2 is naturally resistant to non nucleoside reverse transcriptase inhibitors, the fusion inhibitor enfuvirtide, and has a decreased susceptibility to some of protease inhibitors including the once daily dosing atazanavir [1–3]. A limitation of CCR5 inhibitors use in HIV-2 may be the *in vitro* use of a broad range of coreceptors for this virus, including CCR5 and CXCR4 as well as other alternative coreceptors [4,5]. But some others studies demonstrated that these alternative coreceptors don’t seem to have a major role in viral entry [6–9]. Maraviroc, the only licensed CCR5 antagonist, has been recently shown to be active *in vitro* against HIV-2 R5-tropic viruses [10–12] and a limited number of case reports also showed an early *in vivo* virological response to maraviroc-based treatment [13–15]. Thus, CCR5 inhibitors may represent a new antiretroviral drug class in the HIV-2 limited arsenal as demonstrated *in vitro* with maraviroc.

Cenicriviroc is a new dual CCR5 and CCR2 antagonist. This new compound has completed Phase 2b development for treatment of HIV-1 infection (NCT01338883) [16–18]. Cenicriviroc is administered once daily and is suitable for single-tablet regimen combination products [19]. This point is of a particular interest, because of the complex and twice-daily dosing of maraviroc, and also because of the lack of once-daily dosing therapeutic regimen in HIV-2 due to the natural resistance of HIV-2 to non-nucleoside reverse transcriptase inhibitors and the decrease susceptibility to some protease inhibitors.

The aim of this study was to assess the *in vitro* HIV-2 phenotypic susceptibility to cenicriviroc.

## Patients and Methods

### Study Patients and Virus Stocks

Four R5-tropic, one dual-tropic and one X4-tropic HIV-2 strains, as well as one R5-tropic HIV-1 strain, have been assessed in this study. All these strains were primary clinical isolates issued from distinct HIV-2-infected maraviroc-naïve patients included in the French Agence Nationale de Recherches sur le SIDA et les hépatites virales (ANRS) HIV-2 cohort (CO5) and were isolated by co-cultivation of blood donor, phytohemagglutinin (PHA)-activated peripheral-blood mononuclear cells (PBMC) pool of healthy blood donors as previously described [20]. Written informed consent was provided by all patients at the time of inclusion in the French ANRS CO5 HIV-2 cohort. The ANRS CO5 cohort and substudies has been approved by the French institutional review board “Comité de Protection des Personnes (CPP)—Ile-de-France XI”.

### Tropism Determination

Tropism was assessed for all strains by a tropism phenotypic assay (TPA) recently developed [6,21]. Briefly, this test use Ghost(3) cell lines, expressing CD4+CCR5 or CD4+CXCR4 coreceptors with HIV-2 Long Terminal Repeat-driven Green Fluorescent Protein (GFP) gene activated upon infection. Infection efficiency was quantified by flow cytometric analysis for GFP detection.

### Phenotypic Susceptibility Determination

Phenotypic susceptibility of HIV-2 clinical isolates to cenicriviroc was determined using a slightly adapted ANRS PBMC method [22]. All of the strains have been previously tested for HIV-2 maraviroc susceptibility using the same protocol [4]. All susceptibility assays were performed in RPMI 1640 medium containing 10% (vol/vol) heat-inactivated fetal bovine serum (FBS), 2 mM L-glutamine, and 1000 U/mL penicillin/streptomycin. All PBMC were pooled from three healthy donors and incubated for 3 days in culture medium containing PHA. PHA-activated PBMC were washed and resuspended in medium containing human recombinant interleukin-2 immediately prior to use in an antiviral assay. Drug susceptibility assays were performed in 96-well tissue culture plates. Six-point dilution series of cenicriviroc (final concentrations at 0, 0.05, 0.5, 5, 50 and 500 nM) were prepared in culture medium. PHA-stimulated PBMC were pre-incubated with cenicriviroc at appropriate concentrations during 1 hour before viral infection. Cells were washed and infected with cell-free virus supernatant at 100 median tissue culture infective doses (TCID_50_) for 1 h at 37°C. Cells were subsequently washed once and 2.10^6^ infected PBMC were added to each well of assay plates containing diluted compound. Plates were incubated for 4 days at 37°C in a humidified 5% CO_2_ (vol/vol) atmosphere. Each dilution was tested in quadruplicate. At day 4, viral supernatants were collected and virus replication was quantified by HIV-2 specific viral load measurement using the Generic HIV-2 RNA assay (Biocentric, Bandol, France). All viral supernatants were tested at 100 TCID_50_. The percentage of viral replication inhibition for each concentration of maraviroc was calculated to determine effective concentration 50% (EC_50_) and maximum percentage of inhibition (MPI).

V3 loop direct sequencing was performed on viral supernatants at time of phenotypic assay. No changes, between day 0 and day 4 of the adapted ANRS PBMC phenotypic susceptibility assay, were observed in gp105 V3 loop sequences in all tested HIV-2 clinical isolates.

## Results

**Fig 1:**
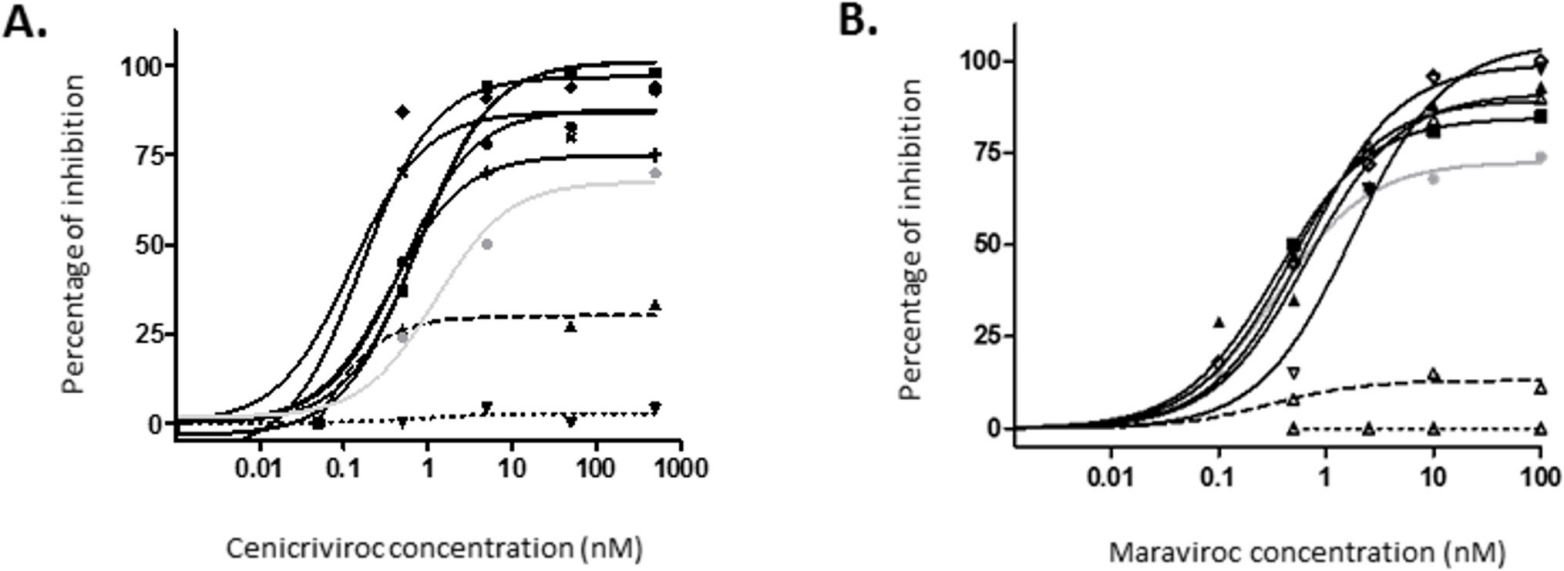
Dose response curves for cenicriviroc-dependent inhibition (A) and maraviroc-dependent inhibition (B). R5, dual and X4-tropic HIV-2 strains are depicted in continuous, dashed and dotted black lines, respectively. HIV-1 R5-tropic strain is depicted using the continuous gray line.

Dose response curves of HIV-2 strains with cenicriviroc are depicted in Fig 1.

Phenotypic susceptibility testing showed, for the four R5-tropic HIV-2 isolates, a median EC_50_ for cenicriviroc of 0.39 nM (0.03, 0.33, 0.45 and 0.98 nM). These results were comparable to those observed in the same conditions with maraviroc with a median EC_50_ of 0.63 nM (respectively 1.13, 0.58, 0.48, 0.68 nM). Concerning MPI, R5-tropic HIV-2 strains presented a median MPI at 94% (94, 94, 93 and 98%), similar to those previously observed for maraviroc using the same protocol with a median MPI at 93% (respectively 93, 90, 82, 100%). The HIV-1 R5-tropic strain presented an EC_50_ at 5.06 nM with a MPI at 70%, similar to those observed for maraviroc at 3.69 nm and 74%.

The dual-tropic and the X4-tropic HIV-2 strains were resistant to cenicriviroc with EC_50_ at >1000 nM, and MPI at 33% and 4%, respectively. Similar results were observed for maraviroc with EC_50_ and MPI above 1000 nM, 12% and above 1000 nM, 0% for the dual-tropic and the X4-tropic, respectively.

## Discussion

In the present study, we demonstrated, for the first time using PBMC assay, that cenicriviroc is active *in vitro* against HIV-2 R5-tropic strains with similar EC_50_ and MPI values to those obtained with maraviroc. HIV-2 EC_50_ for cenicriviroc were also similar to those previously described with HIV-1 (0.29 nM) [16].

Our study presents some limitations. The use of a PBMC phenotypic assay, with detection of viral replication at day 4, has already been depicted as unable to provide MPI at 100% (ref?). The lower MPI observed with this assay can be explained by the competitive fixation of cenicriviroc that allows a low amount of activated PBMC to be infected during the infection step. Thus, the low amount of infected cells is able to produce a non-negligible amount of viral particles at day 4 as observed with HIV-1 and HIV-2 in our previous study concerning maraviroc **[10]**. PBMC assays may have also intrinsic variability due to the PBMC healthy donors’ diversity. This point was taken into account in our assay by the use of a PBMC pool of three healthy donors at each experiment. Despite these limitations, PBMC assay was chosen for evaluation of HIV-2 susceptibility to entry inhibitors because of the possible use of a broad range of accessories coreceptors that is still debated in HIV-2 infection **[4–9]**. Thus, in our assay, the possible use of these accessories coreceptors doesn’t seem to compromise the cenicriviroc *in vitro* efficiency as results were similar to those obtained with HIV-1 for both maraviroc and cenicriviroc.

Cenicriviroc is a novel CCR5 inhibitor in development and clinical studies on cenicriviroc efficacy, tolerance and resistance profile in HIV-2 infection are warranted. However cenicriviroc has demonstrated in this study a similar efficiency *in vitro* against both HIV-2 and HIV-1. This new compound also presents a long half-life supporting once-daily dosing [23]. This point is of particular interest because of the complex dose management and the twice daily dosing of maraviroc, the only CCR5 inhibitor licensed to date. Cenicriviroc may offer new opportunities in the limited HIV-2 therapeutic arsenal.
